# Efficacy and safety of azilsartan medoxomil, an angiotensin receptor blocker, in Korean patients with essential hypertension

**DOI:** 10.1186/s40885-018-0086-4

**Published:** 2018-02-07

**Authors:** Attila Juhasz, Jingtao Wu, Michie Hisada, Tomoka Tsukada, Myung Ho Jeong

**Affiliations:** 1 Takeda Development Center Europe, Ltd., 61 Aldwych, London, WC2B 4AE UK; 2 Takeda Development Center Americas, Inc., One Takeda Parkway, Deerfield, IL USA; 3 Takeda Development Center Asia, Pte. Ltd., 21 Biopolis Road, Nucleos North Tower, Level 4, Singapore, Singapore; 4 Department of Cardiovascular Medicine, Chonnam National University Hospital, 42, Jebong-ro, Dong-gu, Gwangju, 61469 Korea; 5 Present at GE Healthcare, Little Chalfont, UK; 6 Present at Takeda Pharmaceutical Company Ltd., Osaka, Japan

**Keywords:** Hypertension, Azilsartan medoxomil, Angiotensin II receptor antagonist, Blood pressure, Korea

## Abstract

**Background:**

This was a phase 3, randomized, double-blind, placebo-controlled study.

**Methods:**

Adult Korean patients with essential hypertension and a baseline mean sitting clinic systolic blood pressure (scSBP) ≥150 and ≤180 mmHg were randomized to 6-week treatment with placebo (*n* = 65), azilsartan medoxomil (AZL-M) 40 mg (*n* = 132), or AZL-M 80 mg (*n* = 131). The primary endpoint was the change from baseline to week 6 in trough scSBP.

**Results:**

The least-squares mean (standard error) change from baseline in trough scSBP in the placebo, AZL-M 40-mg, and 80-mg groups at week 6 were − 8.8 (2.00), − 22.1 (1.41), and − 23.7 (1.40) mmHg, respectively (*p* < 0.001 for AZL-M 40 and 80 mg vs placebo). No clinically meaningful heterogeneity in efficacy was observed between subgroups (age, sex, diabetes status) and the overall population. Treatments were well tolerated and adverse events were similar between groups.

**Conclusions:**

Results of this study confirm a positive benefit-risk profile of AZL-M for essential hypertension in Korean adults.

**Trial registration:**

Clinicaltrial.gov; identifier number: NCT02203916. Registered July 28, 2014 (retrospectively registered)

**Electronic supplementary material:**

The online version of this article (10.1186/s40885-018-0086-4) contains supplementary material, which is available to authorized users.

## Background

Hypertension is a leading cause of preventable death in developed nations and of increasing prevalence in developing countries [1–4]. Uncontrolled hypertension greatly increases the risk of cardiovascular disease, cerebrovascular disease, and renal failure [5–7]. Despite the availability of antihypertensive agents with various mechanisms of actions, only 13.8–32.5% of patients globally have adequately controlled hypertension (defined as < 140/90 mmHg), with significant disparities in awareness, treatment and control rates and opposite trends for those between high-income and low-to-mid-income countries [8, 9].

The Korean population has adopted a westernized lifestyle, while life expectancy is rapidly increasing [10]. The westernized lifestyle has led to increased rates of cerebrovascular and cardiovascular disease, which are now the second and third most common causes of death in Korea, respectively [11]. Hypertension is associated with 1249 deaths for every 100,000 persons in Korea [11]. Based on data from the Korean National Health and Nutrition Examination Survey, the overall prevalence of hypertension in the population aged ≥30 years increased from 24.6% in 2007 to 28.5% in 2011 [12]. In 2014, the overall prevalence of hypertension in Koreans was 25.5% among adults aged ≥30 years [12]. A similar trend was seen among older adults (aged ≥65 years), with an increased prevalence between 2007 and 2011 (from 49.3 to 58.4% in men, from 61.8 to 68.9% in women, and from 56.6 to 64.6% overall), and a small decrease detected in 2014 (down to 54.3% in men, 65% in women, and 60.5% overall) [13]. Between 2008 and 2011, less than half (42.9%) of patients in Korea had adequately controlled hypertension [12], compared with the diverse global control rates [8, 9].

Pharmacological treatment of hypertension in Korea includes the use of angiotensin II receptor blockers, angiotensin-converting enzyme inhibitors, alpha blockers, beta blockers, calcium antagonists, and diuretics. These classes of antihypertensive agents are often effective, but side effects such as persistent dry cough (angiotensin-converting enzyme inhibitors), tachycardia (calcium antagonists), adverse lipid metabolism (beta blockers and diuretics), and potential worsening of heart failure (alpha blockers) often limit their use [14]. Angiotensin II receptor blockers are generally considered to have a placebo-like safety profile with fewer specific adverse events (cough) compared with angiotensin-converting enzyme inhibitors [15]. Azilsartan medoxomil (AZL-M), an angiotensin II receptor blocker prodrug, was approved for use in 2011 by both the United States Food and Drug Administration and by the European Medicines Agency. AZL-M has been shown to have a strong antihypertensive effect, predictable pharmacokinetic and metabolic profiles, a prolonged duration of action, and good safety and tolerability profiles when administered to hypertensive patients alone or in combination with other antihypertensive drugs [16–19].

Phase 3 studies conducted in the United States, Latin America, and Europe have demonstrated that AZL-M treatment at doses of 20, 40, and 80 mg once daily is well tolerated and effective in reducing high blood pressure in adults with essential hypertension [16, 20, 21]. One of the pivotal phase 3 global registration studies (registered on ClinicalTrials.gov in 2008 [NCT00696241]) comparing AZL-M with olmesartan medoxomil and placebo contributed to the United States Food and Drug Administration and European Medicines Agency approval of AZL-M [16].

Accordingly, this phase 3 study was conducted to evaluate the efficacy and safety of AZL-M during 6 weeks of treatment in Korean adults with essential hypertension. Designed as a bridging study to the global registration study (NCT00696241), only placebo was included as a comparator in this study.

## Methods

This phase 3, multicenter, randomized, parallel-group, double-blind, placebo-controlled study was conducted to evaluate the efficacy and safety of AZL-M 40 and 80 mg once daily in adult Korean patients with essential hypertension and was conducted at 30 study sites in South Korea. This study was registered with ClinicalTrials.gov (NCT02203916) on July 28, 2014. The Institutional Review Boards at each of the 30 study sites were responsible for approval of the clinical study conduct in accordance with ethical principles and the Guidelines of the Declaration of Helsinki, the regulations and guidelines of the International Conference on Harmonisation, Harmonised Tripartite Guideline for Good Clinical Practice, and all applicable local regulations. All patients provided written informed consent prior to screening.

### Study design

**Fig. 1:**
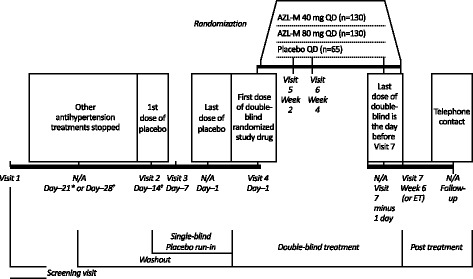
Study design. ^*^Patients taking previous antihypertensive agents were required to participate in a 3-week washout (days − 21 to − 1). ^†^If the patient’s previous antihypertensive treatment included amlodipine or chlorthalidone, then the washout was extended to 4 weeks (days − 28 to − 1). ^‡^Patients who had not received antihypertensive treatment within 28 days prior to screening entered the run-in period as soon as all inclusion and exclusion criteria, including laboratory results, were verified. AZL-M, azilsartan medoxomil; ET, early termination; N/A, not applicable; QD, once daily

The design of this study was modeled upon one of the pivotal phase 3 global registration studies (NCT00696241), that supported approval of AZL-M in approximately 60 countries in Europe, North America, Latin America, the Middle East, and Asia (Fig. 1) [16]. In this study, patients were enrolled and blood pressure was evaluated using sitting clinic blood pressure, whereas the enrollment eligibility and the primary endpoint in the previous global study [16] were determined through ambulatory blood pressure monitoring. Approximately 325 patients (*n* = 130 in each of the two AZL-M arms; *n* = 65 in the placebo arm) were planned for randomization. In the previous global studies, AZL-M 80 mg and 40 mg were demonstrated to provide superior or similar efficacy, respectively, compared with the highest approved doses of the angiotensin II receptor blockers olmesartan medoxomil (40 mg) and valsartan (320 mg) [16, 21], and therefore active controls were not included in this study.

Patients who were previously treated with antihypertensive agents underwent a 3- to 4-week washout that coincided with a 2-week single-blind placebo run-in period. At the start (week − 2) and during (week − 1) the single-blind placebo run-in period, patients returned to the clinic for reassessment of eligibility. Patients who had not received antihypertensive medications within 4 weeks before screening could have entered the run-in period as soon as all inclusion/exclusion criteria, including laboratory parameters, were verified. Patients who qualified for the study were treated with study drug for 6 weeks, with scheduled visits at weeks 2, 4, and 6.

Patients were randomized to receive AZL-M 40 mg once daily, AZL-M 80 mg once daily, or placebo once daily in a 2:2:1 ratio**.** An unbalanced design was chosen based on the global, pivotal phase 3 study (NCT00696241), which also used an unbalanced 2:1 randomization design, and to limit the number of patients treated with placebo-only, given the adequate safety and efficacy profile of AZL-M [16, 20–22]. The study drug blind was maintained throughout the study, and was not to be broken unless information concerning the study drug was essential for the medical treatment of a patient.

### Key inclusion criteria

Adult (≥19 years) male or female Korean patients with baseline mean sitting clinic systolic blood pressure (scSBP) between 150 and 180 mmHg were eligible for inclusion in the study.

### Key exclusion criteria

Patients were excluded if they had post-placebo run-in sitting clinic diastolic blood pressure (scDBP) > 114 mmHg at baseline; secondary hypertension of any etiology; known or suspected unilateral or bilateral renal artery stenosis; history of a major cardiovascular event; poorly-controlled diabetes (HbA1c > 8.0%); estimated glomerular filtration rate < 30 mL/min/1.73 m^2^; alanine aminotransferase level > 2.5× the upper limit of normal; hyperkalemia (defined as serum potassium > upper limit of normal per the central laboratory); a history of hypersensitivity to AZL-M, any of its excipients, or other angiotensin II receptor blockers; or continued use of medication that had a blood pressure effect. Additionally, other patients for whom AZL-M is contraindicated, including pregnant or lactating women, were excluded.

### Blood pressure measurement

For measurements of trough scSBP and scDBP, patients were assessed using the same automated blood pressure device (OMRON HEM-7210, provided by the sponsor) for serial blood pressure measurements (three seated measurements taken a minimum of 2 min apart after cuff deflation). Blood pressure was measured using an appropriately sized cuff (cuff bladder encircling at least 80% of the arm) applied at the upper dominant arm at heart level. Blood pressure measurements were taken approximately 24 h after the previous dose of study drug and prior to dosing or blood collection on the day of clinic visits at day 1 (baseline) and visits at weeks 2, 4, and 6.

### Efficacy and safety endpoints

The primary efficacy endpoint was the change from baseline to week 6 in trough scSBP. Secondary efficacy endpoints included change from baseline to week 6 in trough scDBP, and the percentage of patients who achieved response criteria at week 6 (defined as scDBP < 90 mmHg and/or reduction of ≥10 mmHg from baseline, scSBP < 140 mmHg and/or reduction of ≥20 mmHg from baseline, and reductions in both scDBP and scSBP).

Safety endpoints included adverse events (AEs), vital signs, electrocardiograms, and laboratory parameters. Patients were removed from the study if they experienced hypotension (systolic/diastolic blood pressure < 90/50 mmHg) or were considered for withdrawal from the study if their blood pressure remained elevated (> 180 mmHg systolic and/or > 114 mmHg diastolic) for a 48-h period at any time after screening or randomization.

### Statistical analysis

The full analysis set consisted of all randomized patients who received at least one dose of double-blind study drug. Patients were analyzed according to the treatment group to which they had been randomized. Primary efficacy analysis was performed using the full analysis set. The safety analysis set consisted of all patients who received at least one dose of double-blind study drug.

Patients were analyzed according to the study drug they had received. For the primary efficacy analysis, missing values were imputed using last observation carried forward methodology. Change from baseline in trough scSBP and scDBP was analyzed using an analysis of covariance model, with treatment group as a fixed effect and baseline scSBP or scDBP as a covariate. Estimates of treatment effects and associated *p* values and 95% confidence intervals (CIs) were from the analysis of covariance model. For the primary analysis, the overall type 1 error rate of 0.05 was controlled using the principle of ‘closed’ testing. Under this principle, each of the pairwise comparisons with placebo was conducted at the 0.05 level, with no *p-*value adjustment if the hypothesis “all treatment groups equal” was first rejected at the 0.05 level. Response to treatments based on difference criteria were analyzed using a logistic regression model, with treatment group as a fixed effect and baseline scSBP or scDBP as a covariate; last observation carried forward was used to impute the missing data. The odds ratios of AZL-M doses to placebo and their 95% CIs were estimated based on the model.

Incidence of treatment-emergent AEs was summarized by treatment groups using the safety analysis set. No inferential statistical analyses were performed.

Assuming a standard deviation of 17 mmHg and a 10% dropout rate, a total of 325 enrolled patients was calculated to be sufficient for achieving at least 90% power to detect a difference of 9 mmHg between AZL-M and placebo groups, using a 2-sample t-test of the mean change from baseline in trough scSBP with a 0.05 2-sided significance level. No statistical analysis was planned for the difference between AZL-M 40-mg and AZL-M 80-mg groups.

## Results

### Patient disposition

**Fig. 2:**
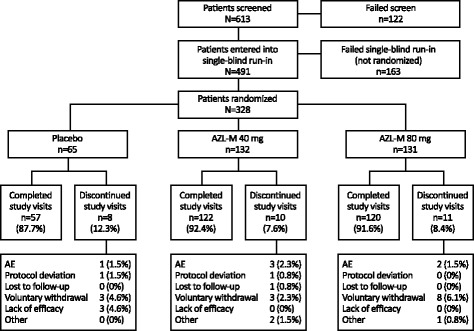
Patient disposition. Patients could have had more than one reason for discontinuation; only the primary reason is presented. AE, adverse event; AZL-M, azilsartan medoxomil

A total of 613 patients were screened. Of these, 491 patients entered the single-blind placebo run-in period, and 328 were randomized into the double-blind period to treatment with placebo (*n* = 65), AZL-M 40 mg (*n* = 132), or AZL-M 80 mg (*n* = 131) (Fig. 2). However, one patient in the AZL-M 80-mg group was randomized twice and excluded from the full and safety analyses sets. Primary reasons for screen failure prior to the single-blind placebo run-in period were patients not meeting entrance criteria (59.8%, 73/122) and voluntary withdrawal (28.7%, 35/122). Primary reasons for non-randomization to receive study drug were patients not meeting entry criteria (89.0%, 145/163) and voluntary withdrawal (8.6%, 14/163).

Overall, 299 patients completed 6 weeks of treatment. A total of 29 patients prematurely discontinued: eight patients (12.3%) in the placebo group, 10 patients (7.6%) in the AZL-M 40-mg group, and 11 patients (8.4%) in the AZL-M 80-mg group. Of 29 subjects who withdrew prematurely, 14 were due to voluntary withdrawal (4.3%, 14/327) and six were due to treatment-emergent AEs (1.8%, 6/327).

### Demographic and baseline characteristics

As required per protocol, all patients were Korean. Patient demographic and baseline characteristics, including age, sex, and body mass index were similar across administration arms **(**Table 1**).** The majority (73.1%) of patients were male and the mean (standard deviation) age was 59.0 (11.0) years; 34.3% (112/327) of patients were ≥65 years. Most (88.4%, 289/327) patients were not classified as diabetic at baseline. There was no statistically significant difference between treatment groups for scSBP (*p* = 0.56) or scDBP (*p* = 0.87) at baseline.

**Table 1:** Patient demographics and baseline characteristics

Parameter	Placebo (*n* = 65)	AZL-M 40 mg (*n* = 132)	AZL-M 80 mg (*n* = 130)	Total (*N* = 327)
Age, mean (SD), years^a^	58.8 (10.2)	59.8 (10.8)	58.3 (11.6)	59.0 (11.0)
< 65 years, *n* (%)	44 (67.7)	82 (62.1)	89 (68.5)	215 (65.7)
≥ 65 years, *n* (%)	21 (32.3)	50 (37.9)	41 (31.5)	112 (34.3)
Sex, *n* (%)				
Male	51 (78.5)	95 (72.0)	93 (71.5)	239 (73.1)
Female	14 (21.5)	37 (28.0)	37 (28.5)	88 (26.9)
Height, mean (SD), cm	166.1 (8.4)	164.8 (9.1)	165.6 (8.0)	165.4 (8.5)
Weight, mean (SD), kg^b^	70.9 (9.9)	71.0 (12.6)	70.3 (13.1)	70.7 (12.3)
BMI, mean (SD), kg/m^2c^	25.64 (2.7)	26.02 (3.3)	25.50 (3.5)	25.74 (3.2)
eGFR, mean (SD), mL/min/1.73 m^2^	85.7 (14.8)	87.4 (18.4)	88.7 (18.4)	87.6 (17.7)
Diabetes status, *n* (%)				
Yes	4 (6.2)	21 (15.9)	13 (10.0)	38 (11.6)
No	61 (93.8)	111 (84.1)	117 (90.0)	289 (88.4)
Concomitant medication, *n* (%)^d^				
Medication continued into double-blind treatment period	26 (40.0)	50 (37.9)	57 (43.8)	133 (40.7)
Initiated use during double-blind treatment period	43 (66.2)	87 (65.9)	89 (68.5)	219 (67.0)
Smoking classification, *n* (%)				
Never smoked	29 (44.6)	71 (53.8)	65 (50.0)	165 (50.5)
Ex-smoker	24 (36.9)	39 (29.5)	39 (30.0)	102 (31.2)
Current smoker	12 (18.5)	22 (16.7)	26 (20.0)	60 (18.3)
scSBP, mean (SD), mmHg^e^	158.8 (7.4)	158.9 (8.0)	160.1 (7.4)	159.4 (7.6)
scDBP, mean (SD), mmHg^f^	94.3 (11.0)	93.1 (9.6)	93.2 (9.5)	93.4 (9.8)

*AZL-M* azilsartan medoxomil, *BMI* body mass index, *eGFR* estimated glomerular filtration rate, *scSBP* sitting clinic systolic blood pressure, *scDBP* sitting clinic diastolic blood pressure, *SD* standard deviation

^a^Age at date of signing informed consent form. ^b^Weight was measured before the first dose of double-blind study drug. ^c^BMI was calculated from the weight taken before the first dose of study drug and height taken at screening. ^d^No clinically meaningful differences were observed between treatment groups in the percentages of patients taking concomitant medications. ^e^No statistical difference in scSBP was observed between treatment groups *(p* = 0.56). ^f^No statistical difference in scDBP was observed between treatment groups *(p* = 0.87)

### Efficacy

**Fig. 3:**
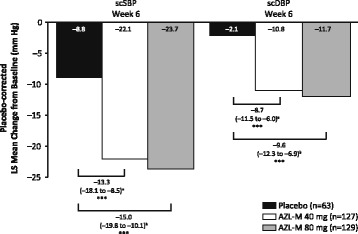
Least squares mean change from baseline to week 6 in trough sitting clinic systolic blood pressure and sitting clinic diastolic blood pressure. ^a^The LS mean difference in change from baseline (95% CI) in AZL-M 40 mg versus placebo (mm Hg [95% CI]). ^b^The LS mean difference in change from baseline (95% CI) in AZL-M 80 mg versus placebo (mm Hg [95% CI]). ***p < 0.001 compared with placebo. Overall treatment effect is statistically significant at 0.05 at all visits for both trough scSBP and scDBP. AZL-M, azilsartan medoxomil; CI, confidence interval; LS, least squares; scDBP, sitting clinic diastolic blood pressure; scSBP, sitting clinic systolic blood pressure

The overall treatment group effect was statistically significant for both changes in the primary endpoint scSBP and secondary efficacy endpoint scDBP from baseline to week 6 (*p* < 0.001 for both). For the primary endpoint, least squares (LS) mean reductions from baseline in trough scSBP to week 6 was 8.8 mmHg, 22.1 mmHg, and 23.7 mmHg in the placebo, AZL-M 40-mg, and AZL-M 80-mg groups, respectively. Relative to placebo, LS mean differences in scSBP were − 13.3 mmHg (95% CI –18.1 to − 8.5) in the AZL-M 40-mg group and − 15.0 mmHg (95% CI –19.8 to − 10.1) in the AZL-M 80-mg group (*p* < 0.001 for both) (Fig. 3).

For the secondary efficacy endpoint, the LS mean reduction in baseline trough scDBP to week 6 was 2.1 mmHg, 10.8 mmHg, and 11.7 mmHg in the placebo, AZL-M 40-mg, and AZL-M 80-mg groups, respectively. LS mean differences for scDBP relative to placebo were − 8.7 mmHg (95% CI –11.5 to − 6.0) in the AZL-M 40-mg group and − 9.6 mmHg (95% CI –12.3 to − 6.9) in the AZL-M 80-mg group (*p* < 0.001 for both) (see Fig. 3).

**Fig. 4:**
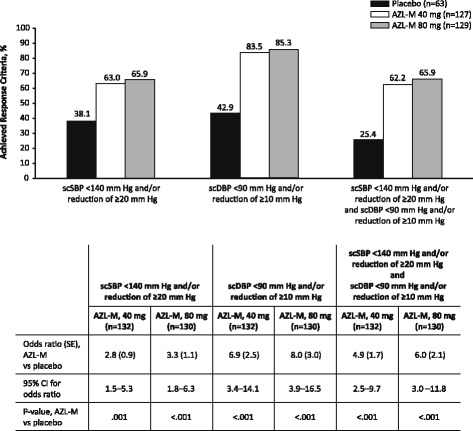
Proportion of patients who achieved sitting clinic systolic blood pressure and/or sitting clinic diastolic blood pressure response at week 6. AZL-M, azilsartan medoxomil; CI, confidence interval; scDBP, sitting clinic diastolic blood pressure; scSBP, sitting clinic systolic blood pressure; SE, standard error

The percentage of patients who achieved scSBP < 140 mmHg and/or a reduction of ≥20 mmHg at week 6 was 38.1% (24/63) in the placebo group compared with 63.0% (80/127) in the AZL-M 40-mg group (odds ratio 2.8, 95% CI 1.5–5.3, *p* = 0.001) and 65.9% (85/129) in the AZL-M 80-mg group (odds ratio 3.3, 95% CI 1.8–6.3, *p* < 0.001) (Fig. 4). For scDBP, 42.9% (27/63) of patients in the placebo group achieved < 90 mmHg and/or a reduction of ≥10 mmHg at week 6 compared with 83.5% (106/127) in the AZL-M 40-mg group (odds ratio 6.9, 95% CI 3.4–14.1, *p* < 0.001) and 85.3% (110/129) in the AZL-M 80-mg group (odds ratio 8.0, 95% CI 3.9–16.5, *p* < 0.001) (see Fig. 4). The percentage of patients who achieved the target response in both scSBP and scDBP was 25.4% (16/63) in the placebo group compared with 62.2% (79/127) in the AZL-M 40-mg group (odds ratio 4.9, 95% CI 2.5–9.7, *p* < 0.001) and 65.9% (85/129) in the AZL-M 80-mg group (odds ratio 6.0, 95% CI 3.0–11.8, *p* < 0.001) (see Fig. 4).

**Fig. 5:**
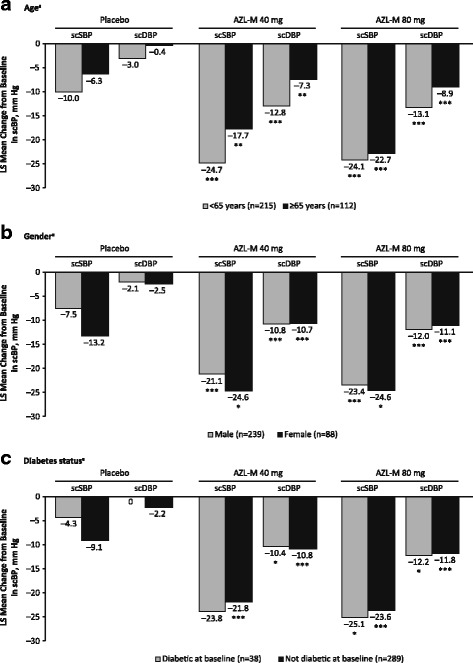
Least squares mean change from baseline to week 6 in trough sitting clinic systolic blood pressure and sitting clinic diastolic blood pressure by (**a**) age, (**b**) sex, and (**c**) diabetes status. *p < 0.05 compared with placebo; **p < 0.01 compared with placebo; ***p < 0.001 compared with placebo. AZL-M, azilsartan medoxomil; LS, least squares; scDBP, sitting clinic diastolic blood pressure; scSBP, sitting clinic systolic blood pressure

Relative to placebo, clinically meaningful changes in scSBP and/or scDBP at week 6 from baseline were observed for subgroups of patients based on patient age, sex, and diabetes status (Fig. 5a to c).

After 6 weeks of treatment, the LS mean difference of AZL-M from placebo on scSBP in patients < 65 years of age was − 14.7 (95% CI –20.5 to − 8.9; *p* < 0.001) for the AZL-M 40-mg group and − 14.1 (95% CI –19.8 to − 8.4; *p* < 0.001) for the AZL-M 80-mg group (Fig. 5a). For patients ≥65 years, the LS mean difference in scSBP was − 11.4 (95% CI –19.9 to − 2.9; *p* = 0.009) in the 40-mg group and − 16.4 (95% CI –25.1 to − 7.7; *p* < 0.001) in the 80-mg group. For patients aged < 65 years, the LS mean difference in scDBP of AZL-M compared with placebo was − 9.9 (95% CI –13.3 to − 6.4; *p* < 0.001) for the AZL-M 40-mg group and − 10.1 (95% CI –13.5 to − 6.7; *p* < 0.001) for the AZL-M 80-mg group. The LS mean difference in scDBP for patients aged ≥65 years was − 6.9 (− 11.3 to − 2.5; *p* = 0.002) for the AZL-M 40-mg group and − 8.5 (− 13.0 to − 4.0; *p* < 0.001) for the AZL-M 80-mg group.

For male patients, the LS mean difference at week 6 in scSBP and scDBP was − 13.6 (95% CI –19.1 to − 8.0; *p* < 0.001) and − 8.7 (95% CI –12.0 to − 5.4; *p* < 0.001), respectively, for the AZL-M 40-mg group, and − 15.9 (95% CI –21.4 to − 10.3; *p* < 0.001) and − 9.8 (95% CI –13.1 to − 6.5; *p* < 0.001) for the 80-mg AZL-M group (Fig. 5b). In female patients, the overall treatment effect was significant for scDBP (*p* = 0.005), but not for scSBP (*p* = 0.113). However, a clinically meaningful effect of AZL-M was observed in female patients in both the AZL-M 40-mg group for scSBP (LS mean − 11.4 [95% CI –21.9 to − 1.0]; *p* = 0.033) and scDBP (LS mean − 8.2 [95% CI –12.9 to − 3.5]; *p* < 0.001) and the AZL-M 80-mg group for scSBP (LS mean − 11.4 [95% CI –21.7 to − 1.1]; *p* = 0.031) and scDBP (LS mean − 8.6 [95% CI –13.2 to − 3.9]; *p* < 0.001) (see Fig. 5b).

For patients with diabetes, the overall treatment effect on scDBP was statistically significant (*p* = 0.015), but not for scSBP (*p* = 0.105). However, the LS mean difference of AZL-M compared with placebo at week 6 on scSBP was significant at 80 mg (LS mean − 20.8 [95% CI –41.5 to − 0.1]; *p* = 0.049); the LS mean difference for 40 mg is − 19.5 (95% CI –39.2 to 0.2; *p* = 0.052) (Fig. 5c). For scDBP, the LS mean difference was − 10.4 (95% CI –19.6 to − 1.2; *p* = 0.028) for the AZL-M 40-mg group and − 12.2 (95% CI –21.9 to − 2.5; *p* = 0.015) for the AZL-M 80-mg group. For patients without diabetes, the LS mean difference at week 6 in scSBP and scDBP was − 12.7 (95% CI –17.8 to − 7.7; *p* < 0.001) and − 8.6 (95% CI –11.5 to − 5.7; *p* < 0.001) for the AZL-M 40-mg group, respectively, and − 14.5 (95% CI –19.5 to − 9.5; *p* < 0.001) and − 9.6 (95% CI –12.4 to − 6.7; *p* < 0.001) for the AZL-M 80-mg group (see Fig. 5c). These differences were considered clinically meaningful.

No clinically meaningful heterogeneity was observed between subgroups and the overall treatment population.

### Safety

In the safety analysis set, 62 patients (19.0%, 69/327) had at least one treatment-emergent AE and the percentage of patients with treatment-emergent AEs was similar between the placebo (20.0%, 13/65), AZL-M 40-mg (15.2%, 20/132), and AZL-M 80-mg (22.3%, 29/130) groups (Table 2). No patient died during the study. Overall, nine patients (2.8%, 9/327) had treatment-emergent AEs that were considered by the investigator to be related to study drug. Six patients (1.8%, 6/327) discontinued study drug because of a treatment-emergent AE; one in the placebo group, three in the AZL-M 40-mg group, and two in the AZL-M 80-mg group. Dizziness was the most frequent treatment-emergent AE considered related to study drug (1.8% [6/327] of all patients). Other treatment-emergent AEs considered related to study drug (headache, hypotension, and dyspepsia) were reported in < 1% of the study population **(see** Table 2**).**

**Table 2:** Treatment-emergent adverse events

	Placebo (*n* = 65)		AZL-M 40 mg (*n* = 132)		AZL-M 80 mg (*n* = 130)		Total (*N* = 327)
	Events, *n*	Patients, *n* (%)	Events, *n*	Patients, *n* (%)	Events, *n*	Patients, *n* (%)	Patients, *n* (%)
Total treatment-emergent AEs	19	13 (20.0)	30	20 (15.2)	40	29 (22.3)	62 (19.0)
Related to study drug	1	1 (1.5)	2	2 (1.5)	7	6 (4.6)	9 (2.8)
Not related to study drug	18	12 (18.5)	28	18 (13.6)	33	23 (17.7)	53 (16.2)
Treatment-emergent AE severity							
Mild	16	11 (16.9)	29	19 (14.4)	25	21 (16.2)	51 (15.6)
Moderate	2	1 (1.5)	1	1 (0.8)	14	7 (5.4)	9 (2.8)
Severe	1	1 (1.5)	0	0	1	1 (0.8)	2 (0.6)
Treatment-emergent AEs leading to discontinuation		1 (1.5)		3 (2.3)		2 (1.5)	6 (1.8)
Serious AEs	0	0	0	0	6	2 (1.5)	2 (0.6)
Deaths		0		0		0	0
Treatment-emergent AEs related to study drug		1 (1.5)		2 (1.5)		6 (4.6)	9 (2.8)
Dizziness		0		1 (0.8)		5 (3.8)	6 (1.8)
Headache		0		1 (0.8)		1 (0.8)	2 (0.6)
Hypotension		0		0		1 (0.8)	1 (0.3)
Dyspepsia		1 (1.5)		0		0	1 (0.3)

*AE* adverse event, *AZL-M* azilsartan medoxomil

Of the patients who experienced a treatment-emergent AE, the majority (82.3%, 51/62) had treatment-emergent AEs that were considered mild in severity. Two patients (0.6%, 2/327) had severe treatment-emergent AEs; one patient experienced a severe headache (1.5%, 1/65) in the placebo group and one patient (0.8%, 1/130) had a tibia fracture in the AZL-M 80-mg group. Overall, two patients (0.6% [2/327]; both in the AZL-M 80-mg group) experienced serious AEs that were deemed unrelated to study drug or study procedure and resolved by the end of the study. The serious AEs included a ligament sprain and patella fracture (due to a traffic accident) in one patient, and a tibia fracture in the other patient.

No clinically meaningful differences were observed between treatment groups in laboratory parameters (including hepatic transaminases, potassium, creatinine, and hemoglobin) (Additional file 1: Table S1), or in vital signs and 12-lead electrocardiogram results. Markedly abnormal creatinine values—defined as > 1.5× the baseline value and above the normal range—were reported in one patient in the AZL-M 80-mg group (baseline value: 85 μmol/L; peak value: 174 μmol/L). This patient completed the study and serum creatinine levels returned to within normal range (103 μmol/L) approximately 2 weeks after week 6, with no signs or symptoms of renal insufficiency. Markedly abnormal uric acid values—defined as > 625 μmol/L in males and > 506 μmol/L in females—were also reported for two (1.5%) patients in the AZL-M 40-mg group only; neither patient had a history of gout.

## Discussion

This was the first phase 3 study to examine the effects of AZL-M, a new angiotensin II receptor blocker, in a Korean population. The results of this study showed that AZL-M, at both 40-mg and 80-mg doses provided clinically meaningful reductions in blood pressure and was well tolerated. The effect of AZL-M 40 mg and 80 mg was reflected via the primary endpoint – change from baseline in scSBP to week 6 relative to placebo (− 13.3 mmHg and − 15.0 mmHg, *p* < 0.001). In addition, a significantly higher percentage of patients in the AZL-M 40-mg (63.0%) and 80-mg (65.9%) groups compared with the placebo group (38.1%) achieved the target scSBP of < 140 mmHg or a reduction of ≥20 mmHg from baseline to week 6. Similar results were also observed for the secondary efficacy endpoints of scDBP and joint reductions in both scSBP and scDBP. Although the overall treatment effect on scSBP was not statistically significant in female patients or in patients with diabetes, the estimated treatment effect for both doses in these two subgroups was considered clinically meaningful. The relatively small number of female patients (*n* = 88/237; 26.9%) and patients with diabetes (*n* = 38/327; 11.6%) enrolled in this study may have resulted in the lack of statistical power to detect differences. Other subgroup analyses for age (< 65 and ≥65), male patients, and patients without diabetes showed both clinically meaningful and statistically significant results.

Results from this study were similar to those from previous phase 3 studies of AZL-M in a global population [16, 21]. In the pivotal phase 3 study by Bakris et al. [16], of 1275 patients with hypertension in a global population, AZL-M 80 mg was found to be more effective in reducing 24-h ambulatory systolic blood pressure compared with olmesartan medoxomil 40 mg, another angiotensin II receptor blocker, with a treatment difference of − 2.1 mmHg (95% CI –4.0 to − 0.1; *p* = 0.038) [16]. In the Bakris et al. [16] study, all three AZL-M doses (20, 40, and 80 mg) were significantly better than placebo in reducing trough scDBP from baseline to week 6 (*p* < 0.001). In the current study, the estimated placebo-adjusted LS mean change in baseline to week 6 showed a more favorable trend of efficacy in both scSBP and scDBP with a greater percentage of patients achieving blood pressure target response than in the study by Bakris et al. [16]. However, these comparisons between studies must be interpreted carefully, as patient eligibility in the study by Barkis et al. [16] was determined by ambulatory blood pressure measurements, which may have resulted in a study population less sensitive to placebo effects and potentially fewer white-coat hypertension compared with the current study. While clinic SBP was used to determine patient inclusion criteria and could therefore have increased the number of patients enrolled with white coat hypertension, the study design included a placebo run-in period to reduce the impact of the placebo effect and ensure baseline hypertension status for all patients. Due to randomization, the average placebo effect was expected to be similar in both placebo and active treatment arms. Further, treatment effect was measured by the placebo-corrected LS mean change from baseline and the placebo response was similar to other angiotensin receptor II blocker monotherapy studies [23–25].

While most studies measure clinic blood pressure, several studies have suggested ambulatory blood pressure measurements are superior in reducing variance and improving reproducibility, therefore reducing sample sizes by up to 50% [26, 27]. Of particular importance is that ambulatory blood pressure may be less affected by a placebo effect compared with clinic blood pressure measurements [28, 29]. As the previous global registration trials enrolled patients based on ambulatory blood pressure measurements, the potential for enrolling white-coat hypertensives was low and placebo effects were minimal compared with the 8.8-mmHg placebo response in this study [16, 21]. Ambulatory blood pressure measurements have several drawbacks, however, including the cost of equipment, extensive staff training, and inconvenience to patients [30]. In the current study, patients were enrolled and evaluated based on their clinic blood pressure. While clinic blood pressure lacks the reproducibility of ambulatory blood pressure measurements, clinic blood pressure is often evaluated in clinical trials and measurement is convenient and easy for both clinicians and patients [30]. The percentage of patients in the placebo group who achieved target scSBP was 38.1%, which was similar to other studies measuring scSBP [31, 32]. Despite this relatively large placebo effect, both doses of AZL-M in this study resulted in statistically significant and clinically meaningful reductions in trough scSBP and scDBP compared with placebo (placebo-corrected reductions in scSBP were 13.3 mmHg and 15.0 mmHg in the AZL-M 40-mg and AZL-M 80-mg groups (*p* < 0.001 for both), respectively). These estimates are similar to those observed in the phase 3 global registration study [16].

In another phase 3 study of 1291 patients with stage 1 or stage 2 hypertension in a US and Latin American population, AZL-M 80 mg showed superior efficacy to valsartan 320 mg and olmesartan medoxomil 40 mg [21]. AZL-M also demonstrated similar efficacy at 40-mg doses compared to olmesartan medoxomil 40 mg with respect to ambulatory systolic blood pressure, but showed a greater reduction in scSBP compared with both olmesartan medxomil and valsartan.

Whereas the majority of studies on hypertension have focused on Western nations, hypertension prevalence is increasing in Asian countries, and it is possible that associations between ethnic and racial differences and antihypertensive treatment safety and efficacy outcomes may exist. In studies comparing south Asian patients to white patients, antihypertensive treatment effects are generally similar between these two groups [33]. However, greater decreases in blood pressure have been associated with a greater reduction in risk of stroke in Asian patients compared with white patients, specifically in patients with diabetes or other associated comorbidities [34]. As such, a target SBP of < 140 mmHg is recommended for Korean patients at risk of stroke or coronary artery disease and in patients with diabetes or chronic kidney disease [14].

The safety and tolerability profile in this study demonstrated a low incidence of treatment-emergent AEs in Korean patients, which is lower than what has been previously reported in studies of AZL-M and is consistent with other antihypertensive medications approved for use in Korea [16, 17, 21, 35–37].

### Limitations

While no differences in efficacy were observed between subgroups and the overall patient population, there were relatively low numbers of female patients (*n* = 88; 26.9%) and patients with diabetes (*n* = 38; 11.6%). AZL-M 40 mg and 80 mg displayed a similar safety profile and showed similar effectiveness in this study, which is consistent with the flat dose response curve evident in angiotensin II receptor blockers [38]. While the majority of patients respond to 40 mg, some will require 80 mg. Further, AZL-M 80 mg is the recommended starting dose in the US, though more comparisons with Asian populations are needed [19].

## Conclusions

The results of this study confirm a favorable benefit-risk profile for AZL-M at doses of 40 mg and 80 mg for adult Korean patients with essential hypertension.

## Additional files

Additional file 1: Table S1. Serum Chemistry Changes from Baseline to Final Visit (Safety Analysis Set). (DOCX 41 kb)

Additional file 2: Text S1. Acknowledgement of Clinical Study Investigators. (DOCX 35 kb)

Additional file 3: Table S2. Institutional Review Board Approvals for the Phase 3 Study (ClinicalTrials.gov, NCT02203916). (DOCX 14 kb)

## Abbreviations

AE: Adverse event
AZL-M: Azilsartan medoxomil
CI: Confidence interval
LS: Least squares
scDBP: Sitting clinic diastolic blood pressure
scSBP: Sitting clinic systolic blood pressure
