# Supplementary material for: Efficacy and safety of azilsartan medoxomil, an angiotensin receptor blocker, in Korean patients with essential hypertension
Source: Clin Hypertens. 2018 Feb 7;24:2. doi: 10.1186/s40885-018-0086-4 (PMC5804062; doi:10.1186/s40885-018-0086-4)
Supplement: Supplementary file 1 — Serum Chemistry Changes from Baseline to Final Visit (Safety Analysis Set). (DOCX 41 kb) [file 40885_2018_86_MOESM1_ESM.docx]

**Additional file 1**

**Table S1.** Serum Chemistry Changes from Baseline to Final Visit (Safety Analysis Set)

| Serum Chemistry Test | Placebo  N=65 | | AZL-M, 40 mg  N=132 | | AZL-M, 80 mg  N=130 | |
| --- | --- | --- | --- | --- | --- | --- |
|  | N | Mean (SD) | N | Mean (SD) | N | Mean (SD) |
| ALT (U/L) |  |  |  |  |  |  |
| Baseline | 65 | 23.8 (8.6) | 132 | 25.4 (12.9) | 130 | 24.4 (13.4) |
| Final visit | 65 | 24.3 (9.0) | 130 | 28.2 (20.1) | 130 | 25.6 (12.8) |
| Change | 65 | 0.4 (7.1) | 130 | 2.9 (12.5) | 130 | 1.1 (11.1) |
| AST (U/L) |  |  |  |  |  |  |
| Baseline | 65 | 24.0 (5.2) | 132 | 25.9 (14.3) | 130 | 24.9 (9.9) |
| Final visit | 65 | 23.9 (5.9) | 130 | 27.8 (27.5) | 130 | 25.7 (9.2) |
| Change | 65 | 0.0 (4.8) | 130 | 1.9 (15.5) | 130 | 0.8 (6.7) |
| Alkaline phosphatase (U/L) |  |  |  |  |  |  |
| Baseline | 65 | 67.4 (15.6) | 132 | 71.4 (19.4) | 130 | 67.8 (18.5) |
| Final visit | 65 | 67.6 (13.0) | 130 | 71.4 (17.8) | 130 | 67.3 (19.0) |
| Change | 65 | 0.2 (9.4) | 130 | 0.1 (8.8) | 130 | –0.6 (8.6) |
| Total bilirubin (μmol/L) |  |  |  |  |  |  |
| Baseline | 65 | 12.0 (5.5) | 132 | 11.5 (5.1) | 130 | 11.0 (4.3) |
| Final visit | 65 | 11.5 (4.8) | 130 | 10.6 (4.6) | 130 | 10.1 (4.2) |
| Change | 65 | –0.5 (4.2) | 130 | –0.9 (3.8) | 130 | –0.8 (3.8) |
| Creatinine kinase (U/L)^a^ |  |  |  |  |  |  |
| Baseline | 65 | 133.4 (111.6) | 132 | 129.8 (99.0) | 130 | 119.5 (95.7) |
| Final visit | 65 | 127.1 (74.8) | 130 | 126.1 (67.7) | 130 | 114.7 (65.0) |
| Change | 65 | –6.3 (78.9) | 130 | –4.1 (75.8) | 130 | –4.9 (91.8) |
| Creatinine (μmol/L) |  |  |  |  |  |  |
| Baseline | 65 | 78.2 (14.2) | 132 | 76.7 (18.8) | 130 | 76.0 (17.7) |
| Final visit | 65 | 77.2 (16.4) | 130 | 77.2 (19.6) | 130 | 77.4 (19.5) |
| Change | 65 | –1.0 (8.2) | 130 | 0.3 (6.8) | 130 | 1.4 (10.8) |
| Glucose (mmol/L) |  |  |  |  |  |  |
| Baseline | 65 | 5.8 (1.0) | 132 | 5.9 (1.0) | 130 | 5.8 (0.9) |
| Final visit | 65 | 5.8 (1.1) | 130 | 5.9 (1.1) | 130 | 5.8 (0.9) |
| Change | 65 | 0.0 (1.0) | 130 | 0.0 (0.8) | 130 | 0.1 (0.7) |
| Potassium (mmol/L) |  |  |  |  |  |  |
| Baseline | 65 | 4.4 (0.4) | 132 | 4.4 (0.4) | 130 | 4.4 (0.4) |
| Final visit | 65 | 4.4 (0.4) | 130 | 4.5 (0.3) | 130 | 4.6 (0.3) |
| Change | 65 | 0.0 (0.3) | 130 | 0.1 (0.3) | 130 | 0.1 (0.4) |
| Sodium (mmol/L) |  |  |  |  |  |  |
| Baseline | 65 | 142.5 (2.5) | 132 | 142.4 (2.1) | 130 | 142.6 (2.2) |
| Final visit | 65 | 142.50 (2.1) | 130 | 142.5 (2.1) | 130 | 142.4 (2.4) |
| Change | 65 | 0.0 (2.6) | 130 | 0.1 (2.4) | 130 | –0.2 (2.6) |
| Uric acid (μmol/L)^b^ |  |  |  |  |  |  |
| Baseline | 65 | 365.8 (78.5) | 132 | 343.1 (90.6) | 130 | 349.7 (81.5) |
| Final visit | 65 | 360.6 (84.5) | 130 | 353.8 (86.6) | 130 | 358.9 (84.7) |
| Change | 65 | –5.2 (53.1) | 130 | 9.6 (45.2) | 130 | 9.2 (55.0) |
| Total cholesterol (mmol/L) |  |  |  |  |  |  |
| Baseline | 65 | 5.0 (1.1) | 132 | 5.0 (1.0) | 130 | 4.9 (0.9) |
| Final visit | 62 | 5.0 (1.0) | 128 | 5.0 (0.9) | 124 | 5.0 (0.9) |
| Change | 62 | 0.0 (0.8) | 128 | 0.0 (0.7) | 124 | 0.1 (0.7) |
| HDL (mmol/L) |  |  |  |  |  |  |
| Baseline | 65 | 1.4 (0.4) | 132 | 1.4 (0.4) | 130 | 1.4 (0.4) |
| Final visit | 62 | 1.4 (0.4) | 128 | 1.4 (0.4) | 124 | 1.4 (0.4) |
| Change | 62 | 0.0 (0.2) | 128 | 0.0 (0.2) | 124 | 0.0 (0.3) |
| LDL (mmol/L) |  |  |  |  |  |  |
| Baseline | 65 | 2.8 (1.0) | 132 | 2.8 (0.9) | 130 | 2.7 (0.9) |
| Final visit | 62 | 2.7 (1.0) | 128 | 2.8 (0.9) | 124 | 2.7 (0.9) |
| Change | 62 | 0.0 (0.7) | 128 | 0.0 (0.7) | 124 | 0.0 (0.7) |
| Triglycerides (mmol/L) |  |  |  |  |  |  |
| Baseline | 65 | 2.0 (2.0) | 132 | 1.8 (1.4) | 130 | 1.8 (1.0) |
| Final visit | 62 | 2.1 (1.6) | 128 | 2.0 (1.8) | 124 | 1.9 (1.3) |
| Change | 62 | 0.1 (1.7) | 128 | 0.2 (0.9) | 124 | 0.1 (1.2) |
| Hemoglobin (g/L) |  |  |  |  |  |  |
| Baseline | -- | 145.0 | -- | 144.0 | -- | 144.3 |
| Final visit | -- |  | -- |  | -- |  |
| Change | -- | –0.6 | -- | –1.5 | -- | –1.5 |

^a^Markedly abnormal creatinine values—defined as >1.5× the baseline value and above the normal range—were reported in 1 patient in the AZL-M 80-mg group (baseline value: 85 μmol/L; peak value: 174 μmol/L). This patient completed the study and serum creatinine levels returned to within normal range (103 μmol/L) approximately 2 weeks after week 6, with no signs or symptoms of renal insufficiency.

^b^Markedly abnormal uric acid values—defined as >625 µmol/L in males and >506 µmol/L in females—were also reported for 2 (1.5%) patients in the AZL-M 40-mg group only; neither patient had a history of gout.

ALT indicates alanine transaminase; AST, aspartate transaminase; AZL-M, azilsartan medoxomil; HDL, high-density lipoprotein; LDL, low-density lipoprotein; scDBP, sitting clinic diastolic blood pressure; scSBP, sitting clinic systolic blood pressure; SD, standard deviation.
