# Supplementary material for: Efficacy and safety of azilsartan medoxomil, an angiotensin receptor blocker, in Korean patients with essential hypertension
Source: Clin Hypertens. 2018 Feb 7;24:2. doi: 10.1186/s40885-018-0086-4 (PMC5804062; doi:10.1186/s40885-018-0086-4)
Supplement: Supplementary file 2 — Acknowledgement of Clinical Study Investigators. (DOCX 35 kb) [file 40885_2018_86_MOESM2_ESM.docx]

**Additional file 2**

**Text S1.** Acknowledgment of Clinical Study Investigators

The authors would like to thank the following clinical study investigators:

- Jang Ho Bae: Konyang University Hospital
- Sang Hong Baek: The Catholic University of Korea Seoul St. Mary’s Hospital
- Shung-Chull Chae: Kyungpook National University Hospital
- Deok-Kyu Cho: Myongji Hospital
- Dong-Ju Choi: Seoul National University Bundang Hospital
- Hyun Hee Choi: Chuncheon Sacred Heart Hospital
- Kook-Jin Chun: Pusan National University Yangsan Hospital
- Woo-Baek Chung: The Catholic University of Korea, Yeouido St. Mary's Hospital
- Joon Hyung Doh: Inje University Ilsan Paik Hospital
- Bum-Kee Hong: Gangnam Severance Hospital, Yonsei University Health System
- Seung Hwan Han: Gachon University Gil Medical Center
- Sang-Ho Jo: Hallym University Sacred Heart Hospital
- Dae Hyeok Kim: Inha University Hospital
- Moo Hyun Kim: Dong-A University Medical Center
- Chong-Jin Kim: KyungHee University Hospital at Gangdong
- Dong-Soo Kim: Inje University Busan Paik Hospital
- Kee Sik Kim: Daegu Catholic University Medical Center
- Myung-A Kim: Seoul Metropolitan Government Seoul National University Boramae Medical Center
- Sang Wook Kim: Chung-Ang University Hospital
- Weon Kim: KyungHee University Hospital
- Won Ho Kim: Chonbuk National University Hospital
- Young-Kwon Kim: DongGuk University Ilsan Hospital
- SangEun Lee: Seoul National University Hospital (during the conduct of the study); Asan Medical Center (current affiliation)
- Jun-Bean Park: Seoul National University Hospital (sub-investigator)
- Do-Sun Lim: Korea University Anam Hospital
- Dong-Ryeol Ryu: Ewha Womans University Mokdong Hospital
- Seung-Jea Tahk: Ajou University Hospital
- JungHan Yoon: Yonsei University Wonju College of Medicine, Wonju Christian Hospital
