# Supplementary material for: Efficacy and safety of azilsartan medoxomil, an angiotensin receptor blocker, in Korean patients with essential hypertension
Source: Clin Hypertens. 2018 Feb 7;24:2. doi: 10.1186/s40885-018-0086-4 (PMC5804062; doi:10.1186/s40885-018-0086-4)
Supplement: Supplementary file 3 — Institutional Review Board Approvals for the Phase 3 Study (ClinicalTrials.gov, NCT02203916). (DOCX 14 kb) [file 40885_2018_86_MOESM3_ESM.docx]

**Additional file 2**

**Table S2.** Institutional Review Board Approvals for the Phase 3 Study (ClinicalTrials.gov, NCT02203916)

| Site Number | IRB Approval Site | IRB Approval Number |
| --- | --- | --- |
| 4001 | Inha University Hospital IRB | 12-99 |
| 4002 | Dong-A University Hospital IRB | 12-158 |
| 4003 | IRB of The Catholic University of Korea, Seoul St. Mary’s Hospital | KC12MDMT0589 |
| 4004 | IRB, Gachon University Gil Medical Center | GBIRB2840-2012 |
| 4005 | IRB, Kyung Hee University Hospital | KMCIRB1224-01 |
| 4006 | IRB, Chonbuk National University Hospital | CUH2012-08-007 |
| 4007 | IRB, Ajou University Hospital | AJIRB-MED-CT3-12-255 |
| 4008 | IRB, Inje University Busan Paik Hospital | 12-150 |
| 4009 | IRB, Konyang University Hospital | 12-63 |
| 4010 | Chonnam National University Hospital IRB | CNUH-2012_167 |
| 4011 | IRB, Korea University Anam Hospital | AN12149-001 |
| 4012 | IRB, Hallym University Sacred Heart Hospital | 2014-3018 |
| 4013 | IRB, Inje University Ilsan-Paik Hospital | IB-1210-035 |
| 4014 | IRB, Myongji Hospital (site name changed from: IRB, Kwandong University College of Medicine MyongJi Hospital) | 4014-12-089 |
| 4016 | Kyung Hee University Hospital at Gangdong IRB | EXP2013-0428 |
| 4017 | Ewha Womans University Mokdong Hospital IRB | 12-31-07 |
| 4018 | IRB, Seoul National University Bundang Hospital | B-1210-173-003 |
| 4019 | Yonsei University Wonju Severance Christian Hospital (site name changed from Yonsei University Wonju Christian Hospital) | #12-1456 |
| 4020 | IRB, DongGuk University ilsan Hospital | 2014-38 |
| 4021 | IRB, Daegu Catholic University Medical Center | CR-14-056-L |
| 4022 | IRB, Yeungnam University Hospital | YUH-14-0361-M34 |
| 4023 | Chung-Ang University Hospital IRB | C2014095(1291) |
| 4024 | IRB, Hallym University Chuncheon Sacred Heart Hospital | 2014-45 |
| 4025 | IRB, Pusan National University Yangsan Hospital | L-2014-131 |
| 4026 | IRB, Seoul National University Hospital | H-1405-086-581 |
| 4028 | IRB, The Catholic University of Korea Yeouido St.Mary's Hospital | SIRB-00218-003 |
| 4029 | IRB, Gangnam Severance Hospital, Yonsei University Health System | 3-2015-0066 |
| 4030 | Kyungpook National University Hospital IRB | KNUH2015-03-007 |
| 4031 | National Health Insurance Service Ilsan Hospital IRB | NHMC2015-03-012 |
| 4032 | IRB, Seoul Metropolitan Government Seoul National University Boramae Medical Center | 20150722/ 16-2015-108/ 081 |

IRB, Institutional Review Board.
